# Cost per response for abatacept versus adalimumab in rheumatoid arthritis by ACPA subgroups in Germany, Italy, Spain, US and Canada

**DOI:** 10.1007/s00296-017-3739-9

**Published:** 2017-05-30

**Authors:** Laure Weijers, Christoph Baerwald, Francesco S. Mennini, José M. Rodríguez-Heredia, Martin J. Bergman, Denis Choquette, Kirsten H. Herrmann, Giulia Attinà, Carmela Nappi, Silvia Jimenez Merino, Chad Patel, Mondher Mtibaa, Jason Foo

**Affiliations:** 1 Real World Strategy and Analytics, Mapi Group, Houten, The Netherlands; 2 Department of Internal Medicine, Rheumatology Unit, University Hospital, Leipzig, Germany; 3 EEHTA-CEIS, Faculty of Economics, University of Rome ‘Tor Vergata’, Rome Italy and Institute of Leadership and Management in Health, Kingston University, London, UK; 4 Department of Rheumatology, University Hospital of Getafe, Madrid, Spain; 5 Drexel University College of Medicine, Philadelphia, PA USA; 6 Institut de Rhumatologie de Montréal, University of Montreal, Quebec, Canada; 7 Bristol-Myers Squibb, Munich, Germany; 8 Bristol-Myers Squibb, Rome, Italy; 9 Bristol-Myers Squibb, Madrid, Spain; 10 Bristol-Myers Squibb, Princeton, NJ USA; 11 Bristol-Myers Squibb, Quebec, Canada

**Keywords:** Incremental cost analysis, Cost-consequence analysis, Biomarker/prognostic factors, Biologic, Disease-modifying antirheumatic drugs, Rheumatoid arthritis

## Abstract

Rheumatoid arthritis (RA) is a chronic inflammatory disorder leading to disability and reduced quality of life. Effective treatment with biologic DMARDs poses a significant economic burden. The Abatacept versus Adalimumab Comparison in Biologic-Naïve RA Subjects with Background Methotrexate (AMPLE) trial was a head-to-head, randomized study comparing abatacept in serum anti-citrullinated protein antibody (ACPA)-positive patients, with increasing efficacy across ACPA quartile levels. The aim of this study was to evaluate the cost per response accrued using abatacept versus adalimumab in ACPA-positive and ACPA-negative patients with RA from the health care perspective in Germany, Italy, Spain, the US and Canada. A cost-consequence analysis (CCA) was designed to compare the monthly costs per responding patient/patient in remission. Efficacy, safety and resource use inputs were based on the AMPLE trial. A one-way deterministic sensitivity analysis (OWSA) was also performed to assess the impact of model inputs on the results for total incremental costs. Cost per response in ACPA-positive patients favoured abatacept compared with adalimumab (ACR20, ACR90 and HAQ-DI). Subgroup analysis favoured abatacept with increasing stringency of response criteria and serum ACPA levels. Cost per remission (DAS28-CRP) favoured abatacept in ACPA-negative patients, while cost per CDAI and SDAI favoured abatacept in ACPA-positive patients. Abatacept was consistently favoured in ACPA-Q4 patients across all outcomes and countries. Cost savings were greater with abatacept when more stringent response criteria were applied and also with increasing ACPA levels, which could lead to a lower overall health care budget impact with abatacept compared with adalimumab.

## Introduction

Rheumatoid arthritis (RA) is a chronic inflammatory disorder characterized by pain and tenderness caused by swelling of synovial joints that often progresses to destructive joint disease, joint damage and impaired joint function. RA is a major cause of sick leave, work disability and reduced quality of life. Consequently, it places a significant financial burden on national economies. In Europe and North America, RA is associated with substantial direct and indirect costs as well as productivity loss. Lundkvist et al. [1] estimated the total health costs (direct, indirect and informal care) of RA to be approximately €45 billion per year in Europe and €41.6 billion in the US.

Abatacept is a selective T cell co-stimulatory modulator administered subcutaneously once a week (an intravenous preparation is also available). It is included in the list of options for use as a first-line biologic disease-modifying antirheumatic drug (bDMARD) in patients with an inadequate response to conventional DMARD therapy in the American College of Rheumatology (ACR) [2] and European League Against Rheumatism (EULAR) [3] guidelines.

Prior studies have analysed the cost-effectiveness of abatacept versus adalimumab in patients with rheumatoid arthritis for whom methotrexate has been providing insufficient response [4–6]. These studies demonstrated the value of abatacept based on the incremental cost-effectiveness ratio per additional quality-adjusted life year [6] or related health benefits and costs per health gain [4, 5].

The Abatacept versus adaliMumab comParison in bioLogic-naïvE RA subjects with background methotrexate (AMPLE) trial was a 2-year head-to-head trial comparing the efficacy of subcutaneous (SC) abatacept versus SC adalimumab in adults with RA. The results of the AMPLE trial demonstrated the comparable efficacy of abatacept and adalimumab with similar overall efficacy benefits across all disease activity measures [7]. Anti-citrullinated protein antibodies (ACPA) are a known biomarker for RA and disease progression, but their predictive value for treatment outcomes is not known [7]. A meta-analysis indicated that the relationship between ACPA status and response to therapy has not been elucidated yet [8] but is of interest. Recent post hoc analyses of the AMPLE trial showed improved efficacy for patients with higher ACPA titre levels. The effect was observed in both clinical efficacy measures ACR20, 50, 70 and 90 responses, changes in disease activity score in 28 joints using the C-reactive protein level (DAS28-CRP), and in improvements in the health-related quality of life disability index (HAQ-DI) [7].

## Objective

The aim of this study was to evaluate the cost-effectiveness of abatacept relative to adalimumab, both in combination with methotrexate (MTX), in ACPA-positive and ACPA-negative patients with RA from the German, Italian, Spanish, US and Canadian healthcare system perspectives based on data and results performed by post hoc analyses of the AMPLE trial.

## Methods

### Economic model

**Fig. 1:**
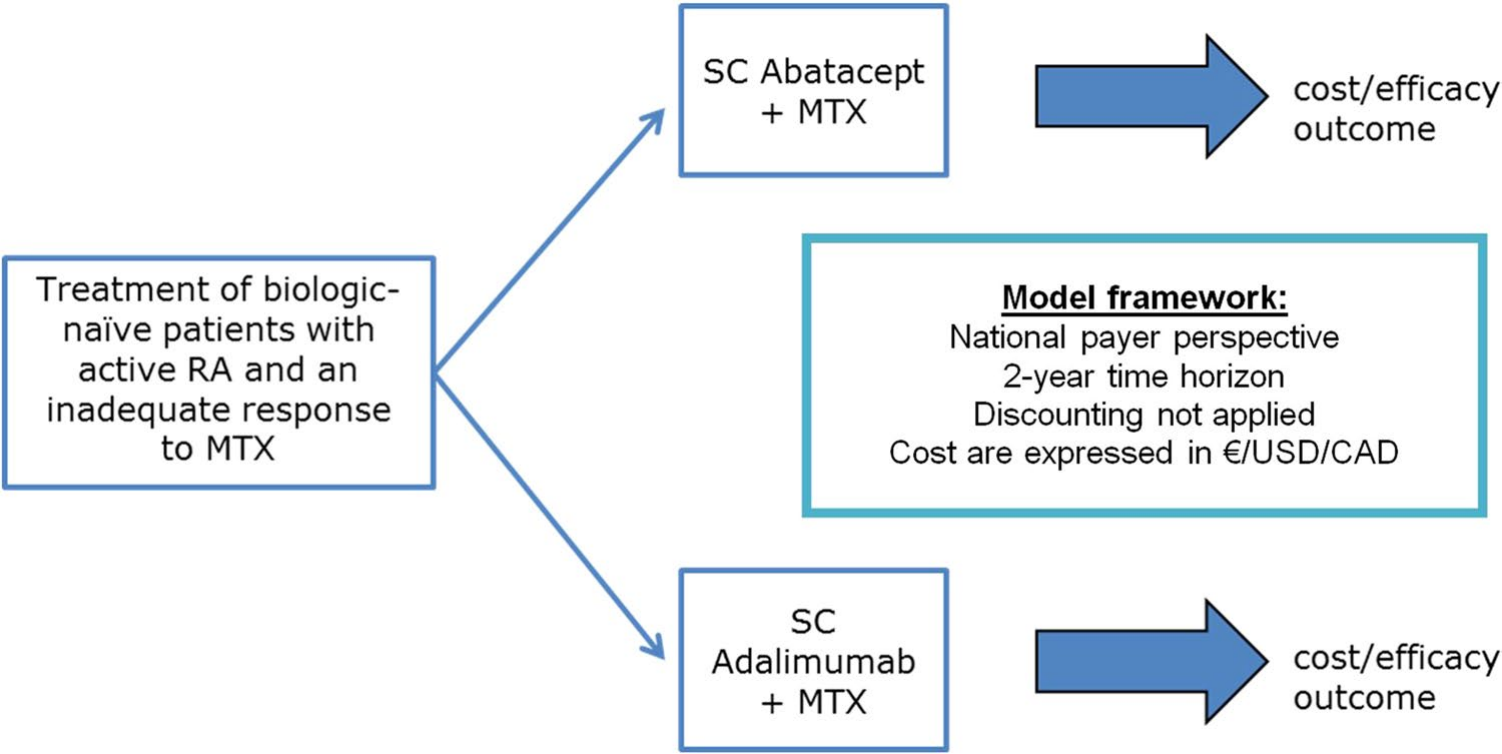
Decision tree structure

A cost-consequence analysis (CCA) from the German, Italian, Spanish, Canadian and US healthcare payer’s perspective was performed. This type of economic evaluation is a variant of a cost-effectiveness analysis (CEA) that presents health-related outcomes alongside costs and subsequently their relative value between alternatives, allowing decision-makers to form their own view of the relative importance of each outcome. In this analysis, direct medical costs associated with the interventions, changes in the response rates, remission rates, and safety profile of patients treated with abatacept and adalimumab were incorporated. A decision analytic model using a deterministic decision tree structure was designed in Microsoft Excel 2010 ( Fig. 1) to calculate monthly costs per responding patient/patient in remission. In line with the AMPLE trial, a time horizon of 2 years was used and given the short time horizon, no discounting was applied.

### Patient population

Patient characteristics at entry into the model were based on baseline characteristics from the AMPLE trial which recruited patients from North and South America [9, 10]. Eligible patients were biologic-naïve adults who had a confirmed diagnosis of RA for 5 years as defined by the ACR revised criteria 1987 [11] despite treatment with MTX. Patients had to have active disease, defined as a score of 3.2 on the DAS28-CRP [12], as well as a history of one or both of the following features: (1) seropositivity for ACPA or rheumatoid factor, and/or (2) an elevated erythrocyte sedimentation rate (ESR) or CRP level. An overview of the baseline characteristics for ACPA-negative and positive patients per quartile had been published previously [10].

### Comparative treatments

Treatment regimens considered in the model were based on the protocol applied in the AMPLE trial as described elsewhere [13]. Briefly, eligible patients were randomized to 125 mg subcutaneous abatacept weekly or 40 mg adalimumab SC bi-weekly. Patients were concomitantly treated with a stable dose of MTX >15 and <25 or >7.5 mg/week in patients with documented intolerance to higher doses. Addition of hydroxychloroquine or sulfasalazine was allowed; addition of other DMARDs, or other investigational or any approved biologic RA therapies other than abatacept and adalimumab during the study was not allowed. Stable low doses of oral corticosteroids (equivalent to <10 mg prednisone daily) were permitted throughout the trial. Nonsteroidal anti-inflammatory drugs (NSAIDs), including aspirin were permitted [9].

### Clinical inputs

Efficacy-related outcomes in the model were determined by the percentage of responding patients according to ACR or HAQ-DI and the percentage of patients in remission according to the DAS28-CRP, the clinical disease activity index (CDAI) and simplified disease activity index (SDAI) (Table 1). The safety-related outcomes were determined by the incidence of frequent adverse events (AEs), serious adverse events (SAEs) (occurring in ≥5% of patients), local injection site reactions (LISRs), malignancies that are not already included as an SAE and autoimmune disorders. These outcomes were largely based on the estimates reported in the clinical study report (CSR) for ACPA-negative and ACPA-positive subgroups in the post hoc analyses of the AMPLE trial.

**Table 1:** Clinical input data for efficacy-related outcomes

Outcome	ACPA-negative		ACPA-positive		ACPA Q1		ACPA Q4	
	Abatacept (*n* = 66) (%)	Adalimumab (*n* = 54) (%)	Abatacept (*n* = 185) (%)	Adalimumab (*n* = 203) (%)	Abatacept (*n* = 42) (%)	Adalimumab (*n* = 55) (%)	Abatacept (*n* = 46) (%)	Adalimumab (*n* = 51) (%)
ACR20 response	47.0	44.4	69.2	66.0	59.5	60.0	78.3	68.6
ACR50 response	34.8	31.5	52.4	53.2	40.5	45.5	63.0	54.9
ACR70 response	19.7	24.1	37.8	33.0	26.2	32.7	43.5	35.3
ACR90 response	7.6	5.6	18.9	10.3	16.7	10.9	17.4	11.8
HAQ-DI response	47.0	29.6	62.2	56.7	57.1	54.5	73.9	60.8
DAS-28 remission	45.1	40.5	55.1	57.5	51.5	56.1	73.2	66.7
CDAI remission	23.5	27.0	38.7	32.1	30.3	31.7	51.2	30.8
SDAI remission	21.6	27.0	36.8	34.0	30.3	36.3	51.2	35.9

### Resource use

Resource use included in the model considered resource utilization items related to RA. Study drug dosage and duration, and concomitant medication duration were obtained from the AMPLE trial [13]. Clinical experts provided input regarding the required resources for the treatment, monitoring and management of the disease and its complications (e.g. daily dosage of concomitant medications, number of outpatient visits, radiographic examinations and routine blood tests). Incidence rates for frequent AEs, SAEs, malignancies, LISRs and autoimmune disorders in either group were also based on the AMPLE trial.

### Costs

Costs in the model included those for study drugs, concomitant drugs and disease monitoring (outpatient visits, radiographic examinations and routine blood tests). The total global costs and per individual cost component were calculated by combining the frequency of resource use with the unit cost per item. Table 2 provides a breakdown of drug costs per country of interest.

**Table 2:** Cost inputs

Drug costs				
Drug	Administration route	Unit	Price	Price/mg
Germany [14]			€	€
Abatacept	SC injection	1 × 125 mg syringe	346.16	2.77
Adalimumab	SC injection	1 × 40 mg syringe	871.89	21.80
MTX (Lantarel^®^)	Oral	1 × 2.5 mg tablet	1.31	0.52
Hydroxychloroquine (Qensyl^®^)	Oral	1 × 200 mg tablet	0.31	0.0015
Sulfasalazine (Sulfasalzin medac^®^)	Oral	1 × 500 mg tablet	0.26	0.00052
Prednisone (Prednison Galen^®^)	Oral	1 × 5 mg tablet	0.16	0.03282
Cyclosporine (Ciclosporin Hexal^®^)	Oral	1 × 100 mg capsule	3.98	0.03983
NSAIDs (Ibuprofen Denk^®^)	Oral	1 × 400 mg tablet	0.16	0.00041
Italy [16]			€	€
Abatacept	SC injection	1 × 125 mg syringe	230.14	1.84
Adalimumab	SC injection	1 × 40 mg/syringe	482.19	12.05
MTX	SC injection	5 × 10 mg (2 ml) syringe	22.15	0.443
Hydroxychloroquine (Plaquenil^®^)	Oral	100 × 200 mg tablets	3.68	0.00018
Sulfasalazine (Salazopyrin^®^)	Oral	100 × 500 mg tablets	9.03	0.00018
Prednisone (Medrol^®^)	Oral	50 × 16 mg tablets	15.51	0.01939
Cyclosporine (Neoral sandimmune^®^)	Oral	30 × 100 mg capsule	74.51	0.02484
NSAIDs (Ibuprofen^®^)	Oral	30 × 100 mg tablets	3.21	0.00107
Spain [15]			€	€
Abatacept	SC injection	1 × 125 mg syringe	194.41	1.56
Adalimumab	SC injection	1 × 40 mg/syringe	475.58	11.89
MTX (Mylan^®^)	SC injection	1 × 25 mg (2 ml) syringe	15.24	0.61
Hydroxychloroquine (Dolquin^®^)	Oral	30 × 200 mg tablets	6.78	0.00113
Sulfasalazine (Salazopyrina^®^)	Oral	50 × 500 mg tablets	2.75	0.00011
Prednisone (Medrol^®^)	Oral	30 × 10 mg tablets	1.48	0.00493
NSAIDs (Ibuprofen^®^)	Oral	40 × 600 mg tablets	1.26	0.00005
US [17]			USD	USD
Abatacept	SC injection	1 × 125 mg syringe	800.82	6.41
Adalimumab	SC injection	1 × 40 mg syringe	1601.05	40.03
MTX	Oral	5 × 10 mg (2 ml) syringe	95.82	1.92
Hydroxychloroquine (Plaquenil^®^)	Oral	100 × 200 mg tablets	638.00	0.0319
Sulfasalazine (Salazopyrin^®^)	Oral	100 × 500 mg tablets	96.14	0.00192
Prednisone (Apo-Prednisone^®^)	Oral	50 × 16 mg tablets	211.36	0.2642
Cyclosporine (Neoral^®^)	Oral	30 × 100 mg capsule	204.51	0.06817
Canada [19]			CAD	CAD
Abatacept	SC injection	1 × 125 mg syringe	366.10	2.93
Adalimumab	SC injection	1 × 40 mg syringe	740.36	18.51
MTX	Oral	1 × 2.5 mg tablets	0.63	0.25
Hydroxychloroquine (Plaquenil^®^)	Oral	1 × 200 mg tablets	0.26	0.0013
Sulfasalazine (Salazopyrin^®^)	Oral	1 × 500 mg tablets	0.18	0.00036
Prednisone (Apo-Prednisone^®^)	Oral	1 × 5 mg tablets	0.02	0.004
Cyclosporine (Neoral^®^)	Oral	1 × 100 mg capsule	3.88	0.0388
NSAIDs (Apo-Ibuprofen^®^)	Oral	1 × 200 mg tablets	0.05	0.00025

Unit costs for routine clinical assessments						
Required resource unit cost		Price				
		Germany €	Italy €	Spain €	US USD	Canada CAD
Radiographic exams	Per session	11.06 [20]	90.38 [22]	23.08 [25]	1125.60 [26]	42.60 [27]
Outpatient visit	Per visit	62.60 [21]	20.66 [23]	100.37 [25]	88.50 [26]	75.00 [27]
Routine blood exams	Per series of tests	7.80 [20]	17.59 [24]	7.59 [25]	103.82 [26]	23.26 [19]

*MTX* methotrexate, *NSAIDs* non-steroidal anti-inflammatory drugs, *na* not applicable

Study drug unit costs and concomitant drug costs were obtained from national databases based on the ex-manufacturer’s price, including mandatory reductions, pay-back, and only for some countries, the transparent discounts [14–19]. The average weight of patients from the AMPLE trial (80.5 kg) was used to calculate the costs of weight-dependent medication.

Disease monitoring costs (routine outpatient follow-up visits, radiographic examinations and routine blood tests) were retrieved from the standard rating scale for outpatient services (EBM, Einheitlicher Bewertungsmaßstab) published by the National Association of Statutory Health Insurance Physicians (KBV-Kassenärztliche Bundesvereinigung), Bock et al. [20, 21] for Germany, the government reimbursement tariffs for hospital stays using the relevant diagnosis-related group (DRG) published by Italian Ministry of Health [22–24] for Italy, the Spanish Ministry of Health [25] for Spain, the Truven Health MarketScan^®^ Commercial Claims and Encounters [26] for the US and the Ontario Ministry of Health and Long Term Care [19, 27] for Canada (Table 2).

Costs for managing frequent AEs and LISRs were based the cost of a general practitioner (GP) visit or a day case. SAEs, malignancies and autoimmune disorders were assumed to require hospitalization. The costs for the management of AEs were retrieved from government reimbursement tariffs for hospital stays using the relevant diagnosis-related group (DRG) in each country.

All costs were expressed in the local currency [2015 euros for Italy, 2015 US dollars (USD) for US, 2015 Canadian dollars (CAD) for Canada and 2016 euros for Germany and Spain]. If necessary, costs were inflated using the relevant country’s consumer price index. In addition, costs were validated by local clinical and economic experts.

### Outcomes of interest

The main outcome measures of interest were the total health benefits and costs per health gain. The costs per health care gain were expressed as the incremental cost per additional responding patient or patient in remission with abatacept versus adalimumab. The achievement of RA clinical response levels was assessed according to ACR20, 50, 70, 90, and HAQ-DI criteria (≥0.3 units). The achievement of remission was assessed in line with the AMPLE trial according to the following thresholds: DAS28-CRP remission, defined as a score of <2.6; ACR/EULAR remission, defined as a CDAI score of ≤2.8 or an SDAI score of ≤3.3. Discontinuation due to any reason, lack of efficacy and safety, risks of SAEs and LISRs were included in the model as safety-related health outcome measures.

### Assumptions

It was assumed that (1) AEs reported as SAEs and LISRs are mutually exclusive events; (2) malignancies as AEs are assumed to be treatment-related and are included in the results for costs; (3) treatment of severe and less severe basal cell carcinoma malignancy is the same; (4) treatment of rash as an AE or LISR is the same; (5) national tariffs applied for unit prices are assumed to include all relevant hospitalization costs, such as inpatient and outpatient visit costs; (6) the list of AEs appearing in more than 5% of patients was taken from the overall AMPLE population; any additional AEs that were seen in subgroups of the data were aggregated under ‘other AEs’. An average AE treatment cost was applied based on the average costs of the listed AEs.

### Analyses

The model simulated 1000 patients (generated from baseline distributions) that were categorized by baseline ACPA quartile and baseline age, sex and HAQ-DI score in line with the AMPLE trial. Changes in HAQ-DI over a lifetime were used to simulate disease progression for each patient. The perspective of the local healthcare system was used and included the costs per outcome per member per month divided by the time horizon of the model (2 years).

The model ran analyses on patients assigned either to abatacept or adalimumab both in combination with MTX according to one of the six ACPA subgroups levels. The cut offs of ACPA levels that defined the subgroups were ACPA-negative: <25 AU/mL, ACPA-positive: ≥25 AU/mL, and ACPA-positive patients divided into four quartiles: Q1: 28–235 AU/mL, Q2: 236–609 AU/mL, Q3: 613–1046 AU/mL, Q4: 1060–4894 AU/mL). These cut offs were selected based on the publication by Sokolove et al. [10], which presents results of the AMPLE trial according to baseline ACPA concentrations.

One-way sensitivity analyses (OWSA) were performed to assess the impact of model inputs on the results for the total incremental costs. All parameters that were represented as a proportion (e.g. percentages) were varied based on their 95% confidence intervals, where available, or by assuming a beta distribution with a standard deviation equal to the mean. Continuous parameters were also varied based on their 95% confidence intervals, where available, or by assuming a triangular distribution with a standard deviation of 30% of the mean. All parameters with a mean of zero were not allowed to vary. Parameters that represent a fixed point, such as time horizon, dosage and duration of bDMARD therapy, concomitant drug prices, cohort size, and patient weight, were not varied as they are not subject to parameter uncertainty.

### Additional analyses-indirect costs

Two additional analyses to incorporate the societal perspective and indirect non-medical costs were performed for Germany and Italy. The model calculates societal costs by combining the cost per HAQ-DI response level, where increasing levels indicate less favourable response, and the associated cost for that category. The German analysis included data on indirect costs obtained from a database study of German patients with RA aged 18–64 [28]. In this study, costs (calculated using the human capital approach) were presented as a function of different HAQ-DI categories to highlight the correlation between work productivity and functional capacity in RA.

The Italian analysis included the costs of work absence and productivity loss due to early retirement. The societal costs by functional capacity were taken from an observational study in Sweden and the UK [29]. Mean costs per patient based on Russo et al. [30] were distributed based on the findings of the observational study by Kobelt et al. that found an association between HAQ-DI response levels and increased costs [29].

The mean cost for work absence was estimated using the number of work days lost per RA employed person multiplied by the daily average income in Italy. The total productivity loss costs were divided by the number of RA patients employed which represents the mean cost per year for an RA patient employed in Italy. The mean costs for early retirement were estimated from the Italian Society Security Agency (INPS) database and referred to inability and invalidity pension. The total pension costs were estimated from Russo et al. [30] and represent the mean annual costs per RA patient receiving a pension in Italy.

## Results

### Health benefits

In general, total health benefits were higher for abatacept in the ACPA-positive and ACPA Q4 subgroups (Table 1). In the ACPA-negative subgroup, total health benefits were higher for abatacept according to all response categories, except for the ACR70. More patients achieved the DAS28-CRP remission criteria with abatacept. Fewer patients discontinued treatment with abatacept for any reason, including efficacy and safety reasons. A lower incidence SAEs (30 versus 93 patients) and LISRs (30 versus 130 patients) were observed with abatacept compared to adalimumab.

In the ACPA-positive subgroup, total health benefits were higher for abatacept using all remission and response outcome criteria, except ACR50 response and remission based on DAS28-CRP. Fewer AEs were observed in patients treated with abatacept compared to adalimumab, as indicated by the difference in patients discontinuing treatment due to safety reasons (43 versus 99 patients) and the total number of patients with SAEs (43 versus 54 patients) and LISRs (49 versus 94 patients).

In the ACPA Q1 subgroup, total health benefits were higher for abatacept with ACR90 and HAQ-DI response. For all remission criteria adalimumab achieved greater health benefits compared to abatacept. Fewer AEs were observed in patients treated with abatacept compared to adalimumab, as indicated by the difference in patients discontinuing treatment due to safety reasons (48 versus 109 patients) and the total number of patients with SAEs (24 versus 73 patients) and LISRs (48 versus 127 patients).

In the ACPA Q4 subgroup, total health benefits were higher for abatacept across all response categories. More patients achieved remission according to DAS28-CRP, CDAI and SDAI criteria with abatacept. Fewer patients discontinued treatment with abatacept for any reason, including efficacy and safety reasons. Abatacept was associated with fewer LISRs compared to adalimumab (22 versus 78 patients). However, a higher incidence SAEs were observed in patients treated with abatacept compared to adalimumab (22 versus 20 patients).

### Costs

When examining costs in Germany and Spain, the total costs for abatacept were lower than adalimumab in both ACPA Q1 and ACPA Q4 patients; however, the difference in costs was lower in ACPA Q4 patients than in ACPA Q1 patients. The incremental costs for ACPA Q1 and ACPA Q4 patients in Germany were −€8,523,373 and −€5,222,805, respectively; and in Spain, −€4,081,075 and −€2,266,701, respectively. The main driver for the increased costs for abatacept in ACPA Q4 patients when compared with ACPA Q1 patients was the cost of acquiring abatacept or adalimumab (approximately 90% of the total costs). However, in both Germany and Spain, abatacept was still cost saving versus adalimumab.

For Italy, the US and Canada, the total costs for abatacept were lower than adalimumab in ACPA Q1 patients but higher in ACPA Q4 patients. The incremental costs for ACPA Q1 and ACPA Q4 patients in Italy were −€610,999 and €362,457, respectively; in the US, −$4,268,568 and $7,693,313, respectively; and in Canada, −$620,436 and $3,157,567, respectively. The main drivers for the increased costs for abatacept in ACPA Q4 patients was the cost of acquiring abatacept or adalimumab in Canada, the costs of managing malignancies in Italy, and the costs of managing SAEs, acquiring abatacept or adalimumab and concomitant medications in the US.

### Incremental costs per health gain

Incremental costs per health gain across all countries according to ACPA status are reported in Table 3. In ACPA-negative patients, the cost per responding/remitting patient when using ACR50, ACR90, HAQ-DI and DAS28-CRP was lower for abatacept compared with adalimumab across all countries. In ACPA-positive patients, results were more consistent with almost all outcomes included in the model showing results in favour of abatacept (except for ACR50 response and DAS28-CRP remission in Italy, US and Canada).

**Table 3:**
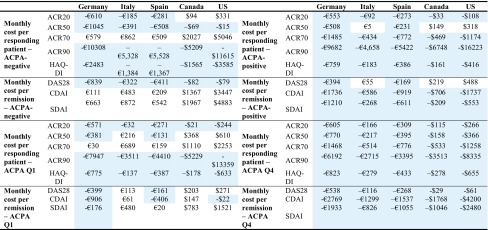
Incremental costs per health gain across all countries (monthly cost per patient on abatacept minus the monthly cost per patient on adalimumab)

In ACPA Q1 patients, the cost per responding/remitting patient using ACR20, ACR90, HAQ-DI was lower for abatacept compared with adalimumab across all countries. In ACPA Q4 patients, all outcomes included in the model showed results in favour of abatacept across all countries. For ACR response outcomes, as the stringency of response criteria increased (ACR20 to ACR90), the cost savings also increased for abatacept relative to adalimumab.

### One-way sensitivity analyses

**Fig. 2:**
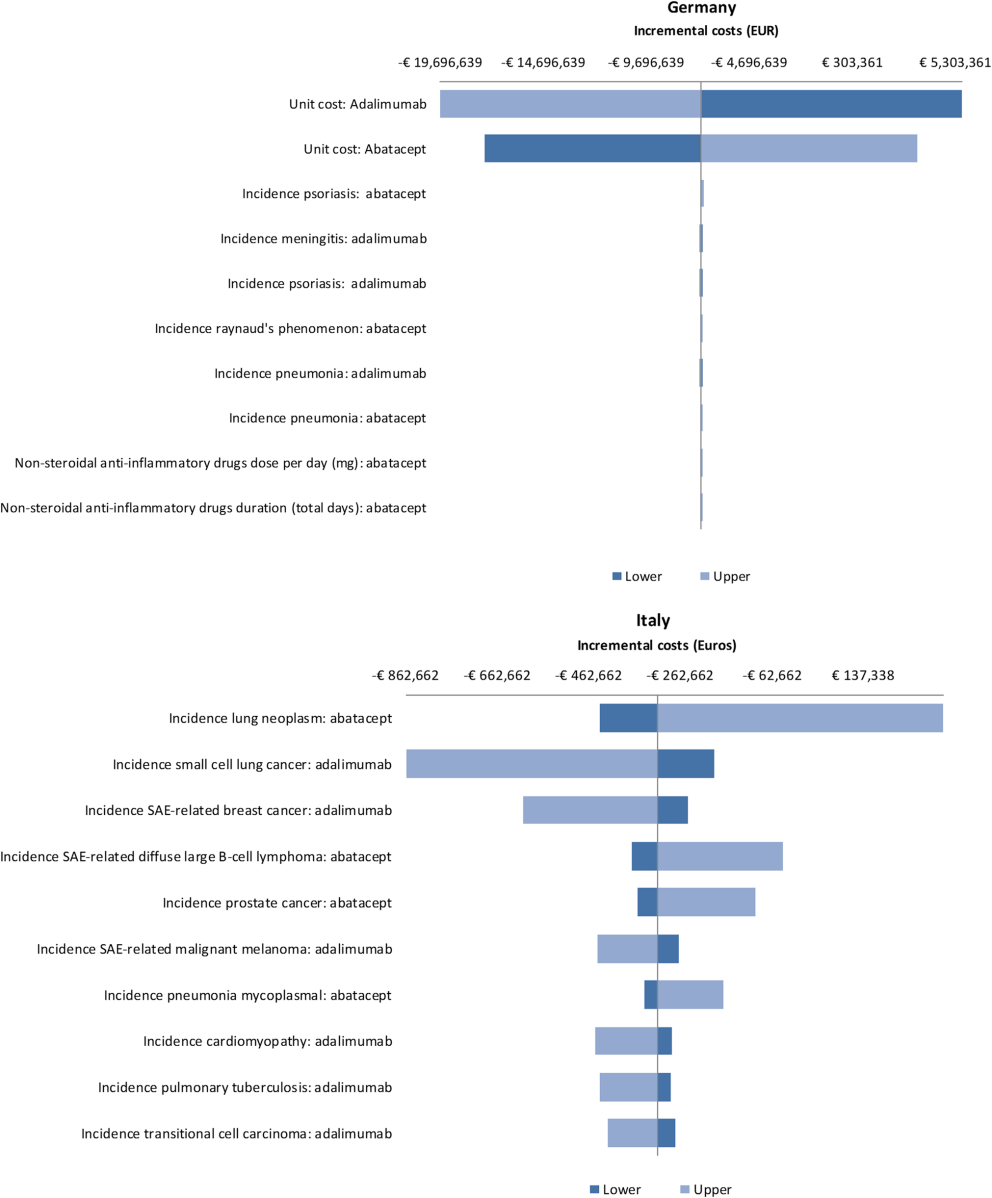

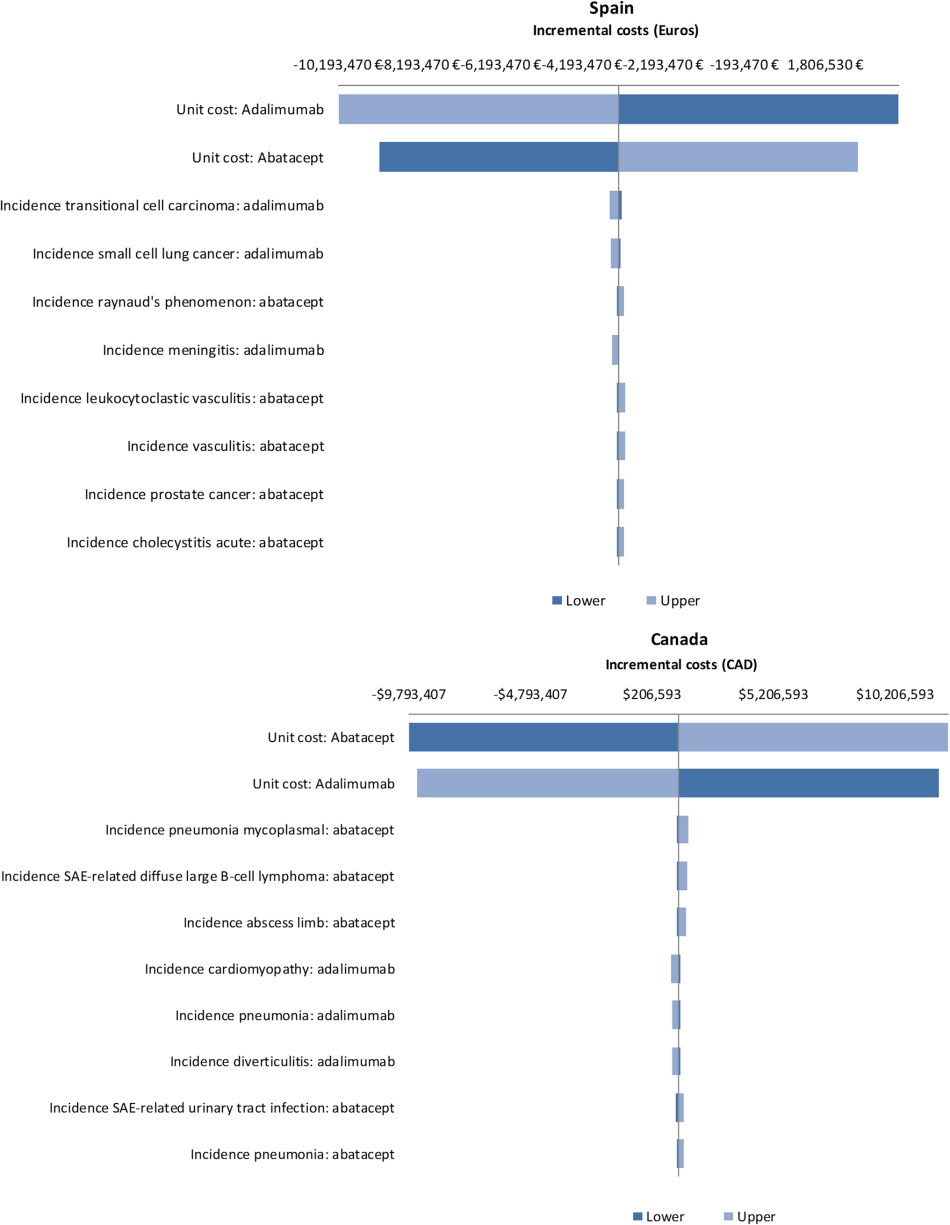

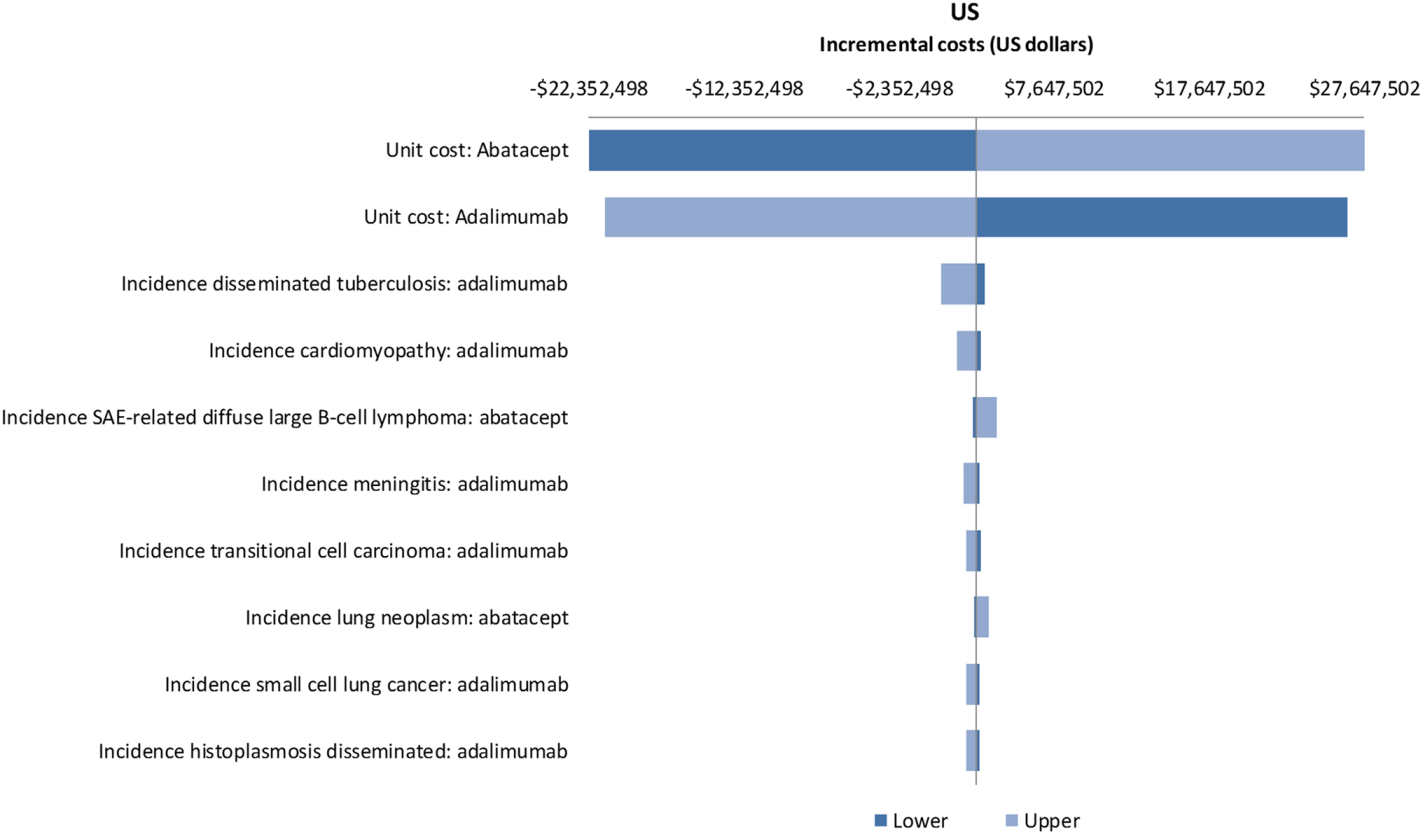
Tornado diagrams depicting the impact of influential parameters on incremental costs for Germany, Italy, Spain, Canada and the US

The results of the OWSA across ACPA subgroups showed that the unit cost of abatacept and the unit cost of adalimumab were the most influential parameters in Germany, Spain, US and Canada. Increasing the unit cost of abatacept or decreasing the unit cost of adalimumab resulted in abatacept no longer being cost saving relative to adalimumab. For all the remaining parameters, abatacept remained cost saving relative to adalimumab. In Italy, for ACPA-negative patients, the incidence of lung cancer and the incidence of malignant melanoma in the adalimumab arm were the two most influential parameters. Increasing the incidence of both of these led to abatacept no longer being cost saving relative to adalimumab. For ACPA-positive patients, the incidence of lung cancer in the abatacept arm and the incidence of small cell lung cancer in the adalimumab arm were the two most influential parameters. However, only increasing the incidence of lung cancer led to abatacept no longer being cost saving relative to adalimumab. For ACPA Q1 patients, the incidence of small cell lung cancer in the adalimumab arm and the incidence of mycoplasmal pneumonia in the abatacept arm were the two most influential parameters. However, abatacept remained cost saving relative to adalimumab even when both of these were increased. For ACPA Q4 patients, the incidence of lung cancer and prostate cancer in the abatacept arm were the two most influential parameters. Decreasing the incidence of both of these resulted in abatacept being cost saving relative to adalimumab. Whilst the incidence of various malignancies was the most influential parameters in Italy, it is worth noting, that the actual incidence of malignancies reported in the AMPLE trial was very low [4]. The results of the OWSA for ACPA-positive patients for all countries are presented in Fig. 2 in the form of a tornado diagram depicting the impact of the ten most influential parameters on the difference in costs between abatacept and adalimumab.

### Additional analyses—indirect costs

Results of additional analysis including societal costs for Germany and Italy are presented in Table 4. In line with the results across all subgroups reported above, cost per responder and cost per patient in remission more consistently favoured abatacept compared to adalimumab in ACPA-positive versus negative patients and ACPA Q4 versus ACPA Q1 patients. The cost-effectiveness of abatacept compared with adalimumab improved when indirect costs are included. These results were consistent across all three countries.

**Table 4:**
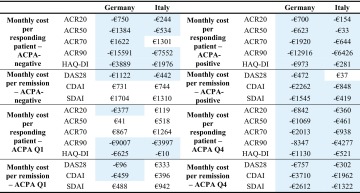
Additional analyses including societal costs—incremental costs per health gain for Germany and Italy (monthly cost per patient on abatacept minus the monthly cost per patient on adalimumab)

## Discussion

The current cost-consequence analysis (CCA) was performed to assess the cost-effectiveness of abatacept in ACPA subgroups from the AMPLE trial from the health care payer perspective of Germany, Italy, Spain, US and Canada. The six subgroups of interest were ACPA-negative patients, ACPA-positive patients and ACPA-positive patients divided into four quartiles according to their ACPA levels. A CCA was chosen, as it presents the results for health benefits as an array of outcomes to allow the decision-makers to form their own view of the relative importance of the health-related outcomes. In the field of RA, where an increasing number of costly biologic treatment options are available to patients, interest in personalized medicine grows. Biomarkers as predictors of response are the subject of an increasing number of studies. ACPA titres are biomarkers that could predict differential responses to biologic treatment over time as has been shown in the AMPLE study.

This CCA demonstrated that the health economic value of abatacept compared to adalimumab across all subgroups and all countries was more pronounced in patients with higher ACPA serum levels. Cost savings were also greater when more stringent response criteria were applied. When indirect costs were included in the model for Germany and Italy, the cost-effectiveness of abatacept compared to adalimumab was further improved. Not considering indirect costs can lead to the potential underestimation of the cost-effectiveness of abatacept compared to adalimumab in patients with RA. The acquisition costs of abatacept and adalimumab were the most influential parameters identified in the OWSA across all subgroups and countries except for Italy.

There are a number of limitations to this study. First of all, the 2-year-time horizon is short considering the young age of onset (i.e. 51 years) and the chronic progressive nature of the disease. Extending the time horizon would require either longitudinal data from the trial, which is not available, or simplifying assumptions for subsequent treatment sequences which is impractical given the various therapy options that are possible in RA [31, 32]. Rather than extend the analysis beyond the available AMPLE data by applying assumptions, it was preferred to perform a more robust analysis relying only on the data from the trial. Nevertheless, economic evaluations usually use short time horizon for the treatment of RA, which is likely a result of the same reasoning made in the present study.

Another limitation pertains to the use of assumptions for the frequency of treatment episodes for AEs that are chronic in nature, such as chronic obstructive pulmonary disease (COPD), malignancies and autoimmune disorders. In addition, the AMPLE was not designed to estimate the efficacy of abatacept SC versus adalimumab SC in ACPA subgroups. A post hoc analysis was performed to elicit the results of these comparisons. Therefore, the number of patients per subgroup is low and the RCT may lack power to estimate significant differences between subgroups. However, even though the trial was not designed to estimate differences between subgroups of patients, Sokolove et al. 2015 [10] found an efficacy pattern in favour of abatacept for quartiles with higher ACPA levels [10].

A third limitation of this study is that the current model estimates the costs associated with common adverse events based on the list of adverse events belonging to the entire AMPLE population. Any additional frequent adverse events occurring that were not in common with the entire AMPLE population were aggregated and added to the model as ‘other adverse events’. An average adverse event cost was then assigned to this category. This approach may have resulted in an over- or underestimation of costs depending on the severity of the adverse events. However, it is assumed that this under- or overestimation has been applied across the board of subgroups and the results of the CCA would not be influenced by this assumption.

Given the increasing number of expensive biologic treatment options, further research is needed in patients with rheumatoid arthritis to help identify subgroups of patients in which treatments are particularly cost-effective. While the quartile thresholds from the AMPLE trial do not exactly match those used in clinical practice; they do suggest subgroups of patients that are likely to benefit the most from abatacept. This knowledge can be used to both benefit patients and reduce the economic impact for national health care systems.

## Conclusion

Across all countries studied the cost per responder and cost per patient in remission was more pronounced for abatacept compared to adalimumab in patients with high ACPA serum levels, a marker of poor prognosis. Cost savings were greater with abatacept when more stringent response criteria were applied and also with increasing ACPA levels. For this patient population, this could lead to a lower overall health care budget impact with abatacept compared to adalimumab in Germany, Italy, Spain, US and Canada and highlights the potential of using ACPA levels to guide prescribers when choosing a bDMARD.

## Abbreviations

ACPA: Anti-citrullinated protein antibodies
AEs: Adverse events
ACR: American College of Rheumatology
bDMARD: Biologic disease-modifying antirheumatic drug
CCA: Cost-consequence analysis
CDAI: Clinical disease activity index
CEA: Cost-effectiveness analysis
COPD: Chronic obstructive pulmonary disease
CRP: C-reactive protein level score
CSR: Clinical study report
DAS28: Disease activity score in 28 joints
DRG: Diagnosis-related group
EULAR: European League Against Rheumatism
ESR: Elevated erythrocyte sedimentation rate
HAQ-DI: Health-related quality of life disability index
LISRs: Local injection site reactions
MTX: Methotrexate
NSAIDs: Non-steroidal anti-inflammatory drugs
OWSA: One-way sensitivity analysis
RA: Rheumatoid arthritis
SAEs: Serious adverse events
SDAI: Simplified disease activity index

## References

[1] Lundkvist J, Kastang F, Kobelt G (2008). The burden of rheumatoid arthritis and access to treatment: health burden and costs. Eur J Health Econ.

[2] Singh JA, Saag KG, Bridges SL, Akl EA, Bannuru RR, Sullivan MC, Vaysbrot E, McNaughton C, Osani M, Shmerling RH, Curtis JR, Furst DE, Parks D, Kavanaugh A, O’Dell J, King C, Leong A, Matteson EL, Schousboe JT, Drevlow B, Ginsberg S, Grober J, St Clair EW, Tindall E, Miller AS, McAlindon T (2016). 2015 American College of Rheumatology Guideline for the Treatment of Rheumatoid Arthritis. Arthritis Rheumatol.

[3] Smolen JS, Landewe R, Breedveld FC, Buch M, Burmester G, Dougados M, Emery P, Gaujoux-Viala C, Gossec L, Nam J, Ramiro S, Winthrop K, de Wit M, Aletaha D, Betteridge N, Bijlsma JW, Boers M, Buttgereit F, Combe B, Cutolo M, Damjanov N, Hazes JM, Kouloumas M, Kvien TK, Mariette X, Pavelka K, van Riel PL, Rubbert-Roth A, Scholte-Voshaar M, Scott DL, Sokka-Isler T, Wong JB, van der Heijde D (2014). EULAR recommendations for the management of rheumatoid arthritis with synthetic and biological disease-modifying antirheumatic drugs: 2013 update. Ann Rheum Dis.

[4] Gaultney J, Benucci M, Iannazzo S, Nappi C, Sion K, Sabater FJ (2015). Trial-based cost-effectiveness of abatacept for rheumatoid arthritis patients in Italy. Expert Rev Pharmacoecon Outcomes Res.

[5] Khanna D, Massarotti E, Rosenblatt L, Budd D, Sabater J, Hebden T (2013) Comparison of cost-efficacy of subcutaneous abatacept versus adalimumab in the treatment of patients with rheumatoid arthritis. Paper presented at the EULAR Annual Congress of Rheumatology, Madrid

[6] Alemao E, Schiff M, Johal S, Al MJ, Rutten-van Molken M (2015) Cost effectiveness analysis of abatacept compared to adalimumab on background methotrexate in biologic-naive RA adult patients by anti-cyclic citrullinated peptide-positive subgroups. Paper presented at the EULAR Annual European Congress of Rheumatology, Rome

[7] Schiff M, Weinblatt ME, Valente R, van der Heijde D, Citera G, Elegbe A, Maldonado M, Fleischmann R (2014). Head-to-head comparison of subcutaneous abatacept versus adalimumab for rheumatoid arthritis: two-year efficacy and safety findings from AMPLE trial. Ann Rheum Dis.

[8] Lv Q, Yin Y, Li X, Shan G, Wu X, Liang D, Li Y, Zhang X (2014). The status of rheumatoid factor and anti-cyclic citrullinated peptide antibody are not associated with the effect of anti-TNFalpha agent treatment in patients with rheumatoid arthritis: a meta-analysis. PLoS One.

[9] Weinblatt ME, Schiff M, Valente R, van der Heijde D, Citera G, Zhao C, Maldonado M, Fleischmann R (2013). Head-to-head comparison of subcutaneous abatacept versus adalimumab for rheumatoid arthritis: findings of a phase IIIb, multinational, prospective, randomized study. Arthritis Rheum.

[10] Sokolove J, Schiff M, Fleischmann R, Weinblatt ME, Connolly SE, Johnsen A, Zhu J, Maldonado MA, Patel S, Robinson WH (2016). Impact of baseline anti-cyclic citrullinated peptide-2 antibody concentration on efficacy outcomes following treatment with subcutaneous abatacept or adalimumab: 2-year results from the AMPLE trial. Ann Rheum Dis.

[11] Arnett FC, Edworthy SM, Bloch DA, McShane DJ, Fries JF, Cooper NS, Healey LA, Kaplan SR, Liang MH, Luthra HS (1988). The American Rheumatism Association 1987 revised criteria for the classification of rheumatoid arthritis. Arthritis Rheum.

[12] Wells G, Becker JC, Teng J, Dougados M, Schiff M, Smolen J, Aletaha D, van Riel PL (2009). Validation of the 28-joint Disease Activity Score (DAS28) and European League Against Rheumatism response criteria based on C-reactive protein against disease progression in patients with rheumatoid arthritis, and comparison with the DAS28 based on erythrocyte sedimentation rate. Ann Rheum Dis.

[13] IM101235. No authors listed. A randomized, head-to-head, single-blind study to compare the efficacy and safety of subcutaneous abatacept versus subcutaneous adalimumab, both with background methotrexate, in biologic-naive subjects with rheumatoid arthritis. Data on file

[14] Taxe L (2016) Available from https://www.lauer-fischer.de/LF/default.aspx?path=WEBAPO-InfoSystem/WEBAPO-Infosystem (Internet). Accessed Feb 2016

[15] The Spanish General Council of Official Colleges of Pharmacists (2015) Medicines database elaborated by the General Council of Pharmacist (*Consejo General de Colegios Oficiales de Farmaceuticos Catalogo de Medicamentos*). Available at https://botplusweb.portalfarma.com

[16] Official Gazette of the Italian Medicines Agency (AIFA). Ex-Factory Price net of GVT measures as AIFA Determination of 03.07.2006 and 27.09.2006

[17] Analysource FD http://www.fdbhealth.com/solutions/analysource-online-drug-pricing-software/. Accessed Aug 2015

[18] (AIFA) AIdF Compendio Farmaceutico Telematico-Banca dati del Farmaco di Farmadati Italia (2014) Available from http://www.agenziafarmaco.gov.it. Accessed June 2016

[19] Ontario Health Insurance Plan (2015) Schedule of Benefits and Fees. Schedule of Benefits for Laboratory Services

[20] National Association of Statutory Health Insurance Physicians (2015) Standard rating scale (Einheitlicher Bewertungsmassstab, EBM). Available from: http://www.kbv.de/html/online-ebm.php. Accessed Feb 2016

[21] Bock JO, Brettschneider C, Seidl H, Bowles D, Holle R, Greiner W, Konig HH (2015). Calculation of standardised unit costs from a societal perspective for health economic evaluation. Gesundheitswesen.

[22] Salute Md (2015) Tariffe delle prestazioni di assistenza specialistica ambulatoriale. Progr. 468

[23] Salute Md (2015). Tariffe delle prestazioni di assistenza specialistica ambulatoriale. Progr..

[24] Salute. Md (2015) Tariffe delle prestazioni di assistenza specialistica ambulatoriale. (DM 1999) 90.62.2., 90.82.5., 90.72.3., 90.44.1, 90.16.3, 90.40.4, 90.37.4, 90.13.3, 90.04.5, 90.09.2, 90.25.5

[25] Ministerio de Sanidad, Servicios Sociales e Igualdad. Available at http://www.msssi.gob.es [Internet]. Accessed Dec 2015

[26] Truven Health Analytics (2015) Cost Inputs for US Adaptation of AMPLE/CCA Model

[27] Schedule of Benefits (2015) Physician Services Under the Health Insurance Act

[28] Huscher D, Mittendorf T, von Hinuber U, Kotter I, Hoese G, Pfafflin A, Bischoff S, Zink A (2015). Evolution of cost structures in rheumatoid arthritis over the past decade. Ann Rheum Dis.

[29] Kobelt G, Lindgren P, Lindroth Y, Jacobson L, Eberhardt K (2005). Modelling the effect of function and disease activity on costs and quality of life in rheumatoid arthritis. Rheumatology (Oxford).

[30] Russo S, Mariani TT, Migliorini R, Marcellusi A, Mennini FS (2015). The economic burden of musculoskeletal disorders on the Italian social security pension system estimated by a Monte Carlo simulation. Reumatismo.

[31] Benucci M, Saviola G, Manfredi M, Sarzi-Puttini P, Atzeni F (2011). Cost effectiveness analysis of disease-modifying antirheumatic drugs in rheumatoid arthritis. A systematic review literature. Int J Rheumatol.

[32] Furneri G, Mantovani LG, Belisari A, Mosca M, Cristiani M, Bellelli S, Cortesi PA, Turchetti G (2012). Systematic literature review on economic implications and pharmacoeconomic issues of rheumatoid arthritis. Clin Exp Rheumatol.

